# Spiritual emptiness and depression among university students: the roles of perceived support and age

**DOI:** 10.3389/fpsyg.2026.1804480

**Published:** 2026-08-12

**Authors:** Lei He, Meng-Yi Huang, Yi-Fei Zhao, Margaret Xi Can Yin, Ding-Hua Tang

**Affiliations:** 1 Student Affairs Office, School of Chemistry and Chemical Engineering, Southeast University, Nanjing, China; 2 School of Humanities, Southeast University, Nanjing, China; 3 Department of Social Work and Social Administration, The University of Hong Kong, Hong Kong, Hong Kong SAR, China; 4 School of Civil Engineering, Southeast University, Nanjing, China

**Keywords:** Chinese university students, depression, perceived support, scale validation, spiritual emptiness

## Abstract

**Introduction:**

Spiritual emptiness and depression are becoming increasingly prevalent among Chinese university students. Perceived social support plays a pivotal role in mitigating both spiritual emptiness and depression. This study investigates interrelationships among these three variables within a sample of Chinese university students.

**Methods:**

A total of 1,266 university students in China, encompassing all gender identities and aged 18–30, completed a questionnaire containing the Multidimensional Scale of Perceived Social Support (MSPSS), the Multidimensional Sense of Emptiness Scale (MSES), and the Patient Health Questionnaire (PHQ- 9). These instruments measured perceived social support, spiritual emptiness, and depression, respectively. The Chinese version of the MSES was validated, and regression analyses and path analyses were performed.

**Results:**

The results demonstrated good reliability and validity of the MSES for assessing spiritual emptiness among Chinese university students, supporting its original three-factor structure. The findings revealed a relatively high incidence of depressive symptoms (26.5%) among the participants. Perceived social support was significantly and negatively correlated with both spiritual emptiness and depression. Additionally, spiritual emptiness partially mediated the relationship between perceived support and depression, while age was found to moderate this relationship.

**Discussion:**

These results suggest that supportive interventions targeting spiritual emptiness and depression should be provided to university students to mitigate these challenges, especially for older students.

## 1 Introduction

### 1.1 Depression among university students

Mental health problems among university students have attracted increasing global attention. This demographic demonstrates a heightened vulnerability to depression, with a detection rate of 24.1% in the 18–24 age group, which is significantly higher than the 4.7–12.3% rate found in other age groups (Psychology China, 2023). Prior research has consistently shown high levels of depressive symptoms among university students. In China, academic pressure, employment uncertainty, and rapid social change further exacerbate psychological distress during the transition to adulthood (Beiter et al., 2015; Chen et al., 2005; Cui et al., 2022; Ibrahim et al., 2013; Auerbach et al., 2018).

Depression is a prevalent and debilitating mental health disorder characterized by persistent low mood, anhedonia, and cognitive and somatic symptoms (Mouchet-Mages and Baylé, 2008; Penninx et al., 2013; Watson et al., 2020). It represents a leading cause of disability worldwide and has substantial impacts on individual functioning and wellbeing (Friedrich, 2017).

### 1.2 Spiritual emptiness as an existential risk factor

Spiritual emptiness is characterized by an inner void, meaninglessness, and lack of relatedness (Ermis-Demirtas et al., 2022). Individuals experiencing this state often report diminished meaning in life, reduced motivation, existential isolation, boredom, and emotional numbness (Ho and Ho, 2007; D’Agostino et al., 2020). In university students, such experiences may contribute to low wellbeing, difficulty in goal-setting, and reduced life satisfaction (Yonker et al., 2012; Debats and Drost, 1995; Steger et al., 2008).

Importantly, spiritual emptiness has been consistently associated with depressive symptoms. Evidence suggests that deficits in meaning and interpersonal connectedness may increase vulnerability to depression (Bonelli et al., 2012; Braam and Koenig, 2019). However, although closely related, spiritual emptiness and depression are theoretically and empirically distinct constructs. The former reflects an existential experience characterized by meaninglessness and relational disconnection, whereas depression is defined by persistent affective, cognitive, and somatic symptoms (Ermis-Demirtas et al., 2022).

Empirical findings further support their discriminant validity. Factor-analytic studies using the Subjective Emptiness Scale (SES) and the Multidimensional Sense of Emptiness Scale (MSES) have shown that emptiness and depression load on separate latent factors, with two-factor models demonstrating better fit than single-factor models (Price et al., 2020; D'Agostino et al., 2021). Differences in associations with externalizing and self-harm-related behaviors also support their distinction (Klonsky, 2008).

### 1.3 Perceived social support

Perceived social support refers to individuals’ subjective perception that emotional and instrumental resources are available from significant others, and it represents a key protective factor against psychological distress (Wethington and Kessler, 1986). Extensive empirical evidence shows that higher perceived social support is associated with lower depressive symptoms among university students (Roohafza et al., 2014; Uchino et al., 2012; Shi, 2021).

Beyond its buffering effect on depressive symptoms, perceived social support is also closely related to psychological resources such as meaning in life (Xue et al., 2023) and belongingness (Baumeister and Leary, 1995). These findings suggest that perceived social support may influence not only affective states but also deeper existential experiences. In particular, it may contribute to individuals’ sense of meaning and interpersonal connectedness, thereby potentially reducing experiences of spiritual emptiness. This suggests that perceived social support may correlate with depressive symptoms via its concurrent associations with existential experiences.

### 1.4 Theoretical framework

This study is primarily grounded in the stress-buffering model of social support, which proposes that perceived social support serves as a critical psychosocial resource that mitigates the adverse effects of stress on psychological wellbeing. According to this model, individuals with higher levels of perceived social support are less likely to experience negative emotional states such as depression, as social resources enhance coping capacity and psychological resilience (Cohen and Wills, 1985). Beyond stress-buffering effects, existential perspectives further suggest that disruptions in meaning and interpersonal relatedness are central contributors to depressive symptoms. Prior research has indicated that deficits in meaning and interpersonal connection are key sources of depressive vulnerability, whereas spiritual resources may serve as protective factors that alleviate depressive severity (Glaw et al., 2017; Sorajjakool et al., 2008). Empirical evidence indicates that meaning in life is negatively associated with depressive symptoms and can counteract core depressive features such as anhedonia and reduced intrinsic motivation (Frankl, 1985; McKnight and Kashdan, 2009; Tian et al., 2025). Building on this perspective, insufficient perceived social support may undermine individuals’ sense of meaning and interpersonal connectedness, thereby contributing to experiences of spiritual emptiness.

### 1.5 The present study

Based on previous literature and the proposed theoretical framework, this study aimed to examine the relationships among perceived social support, spiritual emptiness, and depressive symptoms among Chinese university students. Given that developmental stage may influence the stability of interpersonal resources and coping capacity, it is important to examine whether favorable correlational pattern of perceived social support varies across age groups.

Accordingly, the following hypotheses were proposed:

*H1*: Perceived social support is negatively associated with spiritual emptiness.

*H2*: Spiritual emptiness is positively associated with depressive symptoms.

*H3*: Spiritual emptiness mediates the relationship between perceived social support and depressive symptoms.

*H4*: Age moderates the direct association between perceived social support and depressive symptoms.

**Figure 1:**
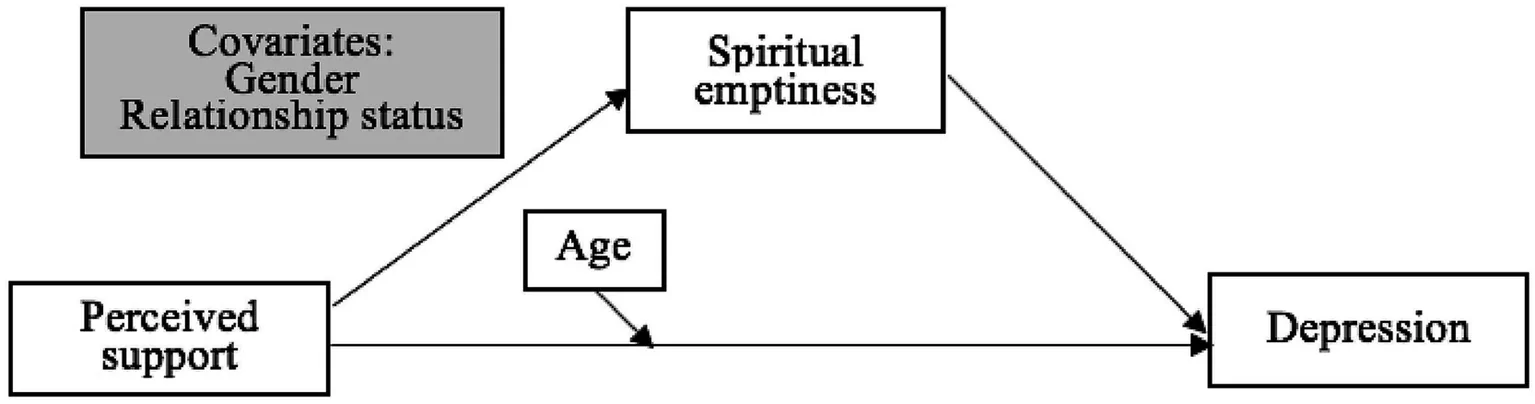
The hypothetical moderated mediation model.

The hypothetical framework for this study is shown in Figure 1.

In addition, prior research has indicated that socio-demographic factors such as age, gender, marital status, and family income may influence perceived social support and depressive symptoms (Snowdon, 2001; Seedat et al., 2009; Gibson et al., 2016; Melchior et al., 2010; Vaingankar et al., 2020). Accordingly, gender and relationship status were included as general demographic covariates, given their established roles in shaping interpersonal resources and psychological wellbeing. Religious belief was further included due to its documented associations with meaning-making processes, spiritual experiences, and depressive symptoms (Koenig, 2012; Garssen et al., 2021). Only-child status was also controlled for given its cultural and developmental relevance in the Chinese context (Falbo and Poston, 1993). These covariates were included to reduce potential confounding and to ensure more precise estimation of the proposed associations.

## 2 Method

### 2.1 Participants

Participants were recruited from March to April 2024 via online and offline open recruitment using posters at seven universities in Nanjing, China. Nanjing was selected as the study setting due to its large and diverse higher-education student population, with over one million students enrolled across a broad range of institutions, including key universities, general public universities, and private institutions (Nanjing Municipal Bureau of Statistics, 2025). This institutional diversity enhances sample heterogeneity and supports the representativeness and generalizability of the study findings.

For online recruitment, study posters containing a brief description of the research and a QR code to an online survey platform were distributed through university student groups. The online survey included an informed consent form followed by the questionnaire. For offline recruitment, printed questionnaires were distributed on campus by trained research assistants at the participating universities, with informed consent obtained prior to participation.

Eligible participants were undergraduate or postgraduate students aged 18–30 years who provided informed consent. Responses were excluded if they were completed in an unrealistically short amount of time, contained substantial missing data, or exhibited obvious response patterns (e.g., selecting the same response option throughout the questionnaire). A total of 1,436 university students completed the questionnaire (1,126 online and 310 via hard copies). After data screening, 170 cases were excluded, and 1,266 valid questionnaires were retained for analysis, yielding an effective response rate of 88.16%.

All respondents participated voluntarily, and their personal information was kept confidential. Consent forms were obtained from all respondents. The study has obtained the approval of Human Research Ethics Committee of Hong Kong University (No. EA210464).

### 2.2 Measures

*Socio-demographic characteristics.* Information on age, gender, only-child status, family monthly income, religious belief, and relationship status was collected, as these factors have been shown to be associated with psychological wellbeing and social support among young adults in previous research (Snowdon, 2001; Seedat et al., 2009; Gibson et al., 2016; Melchior et al., 2010; Vaingankar et al., 2020; Koenig, 2012; Garssen et al., 2021; Falbo and Poston, 1993).

*Perceived support*. Perceived support was measured using the family and friends subscales of the Multi-dimensional Scale of Perceived Social Support (MSPSS) (Zimet et al., 1988). The scale comprises four items evaluating relationships with friends and four items assessing family relationships, rated on a 7-point Likert scale ranging from 1 (very strongly disagree) to 7 (very strongly agree). Total scores range from 8 to 56, with higher scores reflecting greater perceived support. The Chinese version of the MSPSS and its subscales have demonstrated strong reliability and validity, with a Cronbach’s *α* of 0.75 (Zhao et al., 2022).

*Spiritual emptiness.* Spiritual emptiness was measured using the Multidimensional Sense of Emptiness Scale (MSES) (Ermis-Demirtas et al., 2022), a recently developed instrument consisting of 13 items rated on a 5-point scale (1 = none or little of the time true of me; 5 = most or all of the time true of me). The scale includes five items assessing Sense of Inner Emptiness, five items measuring Sense of Absence of Relatedness, and three items evaluating Sense of Meaninglessness. Total scores range from 13 to 65, with higher scores indicating a greater degree of spiritual emptiness. The MSES has shown excellent reliability and validity, with a Cronbach’s *α* of 0.97. For this study, the MSES was translated into Chinese using a forward-backward translation procedure. The initial translation was completed by professional translators, and the Chinese version was subsequently back-translated into English by an independent bilingual translator. The translated versions were reviewed by psychologists, and discrepancies were resolved through discussion to ensure conceptual equivalence between the original and Chinese versions. Prior to the main survey, a pilot study involving approximately 30 students was conducted to evaluate the clarity, comprehensibility, and preliminary reliability of the translated scale. The pilot results indicated excellent internal consistency (Cronbach’s *α* = 0.966). Feedback from the pilot participants indicated that the items were generally clear and understandable. The psychometric properties of the Chinese version were subsequently evaluated among college students in China, as reported in the Results section.

*Depression.* Depression was evaluated using the nine-item Patient Health Questionnaire (PHQ-9) (Spitzer et al., 1999), a widely used tool for assessing the frequency of depressive symptoms. Items are rated on a 4-point Likert scale ranging from 0 (not at all) to 3 (nearly every day), yielding a total score ranging from 0 to 27, with higher scores indicating more severe depressive symptoms. The Chinese version of the PHQ-9 has demonstrated good reliability and validity among university students, with a Cronbach’s *α* of 0.80. A total score of 10 or above has been recommended as the cut-off for clinically significant depressive symptoms (Du et al., 2017).

### 2.3 Data analysis

To evaluate the structure validity of the Chinese version of the MSES, exploratory factor analysis (EFA) and confirmatory factor analysis (CFA) were performed. The total sample (*N* = 1,266) was randomly split into two independent subsamples using R 4.6.1: subsample 1 (*n* = 633) for EFA and subsample 2 (*n* = 633) for CFA, allowing cross-validation of the factor structure. EFA was conducted using the Maximum Likelihood Robust (MLR) estimator with oblique Geomin rotation. Factors with eigenvalues greater than 1.0 were retained, and factor loadings ≥ 0.40 were considered acceptable (Dalawi et al., 2025). Cronbach’s *α* was used to assess internal consistency reliability, with values above 0.70 indicating acceptable reliability (Kim et al., 2016). CFA was subsequently conducted to evaluate the factor structure identified in the EFA. Model fit was assessed using the Comparative fit index (CFI), Tucker-Lewis Index (TLI), Root Mean Square Error of Approximation (RMSEA), and Standardized Root Mean Square Residual (SRMR). A model was deemed to have good fit if the CFI and TLI values were greater than 0.95, and the RMSEA and SRMR values were below 0.08 (Hooper et al., 2008). Both EFA and CFA analyses were conducted using Mplus 7.0.

Continuous data were expressed as mean ± standard deviation (SD), while categorical data were presented as frequencies and percentages, and categorical demographic covariates were dummy coded prior to regression and moderated mediation analyses. Gender differences in sociodemographic information and study variables were examined using one-way analysis of variance (ANOVA) or chi-square tests, as appropriate. Relationships between the study variables were assessed using two-tailed Pearson’s correlation tests. Based on the correlation results, regression models were employed to evaluate the associations between perceived support, spiritual emptiness, and depression. Model 1 tested the association of six control factors with the three studied variables: age (continuous), gender (male was set as the reference group; dummy 1 = 1 for female, 0 otherwise; dummy 2 = 1 for other genders, 0 otherwise), relationship status (0 = single, 1 = not single), family income (the lowest tier below 3,000 RMB as reference group; dummy1 = 1 for 3,000–9,999; dummy2 = 1 for 10,000–49,999; dummy3 = 1 for above 50,000), religion (0 = have a religion, 1 = have no religion), and only-child status (0 = yes, 1 = no). Model 2 included perceived support as an independent variable based on model 1. Model 3 examined the relationship of the six control factors, perceived support, and spiritual emptiness with depressive symptoms. The hypothesized moderated mediation model was tested using Model 5 from Hayes’ PROCESS macro (Hayes, 2017). Variables were mean-centered and a bootstrap sample of 5,000 was used with 95% confidence interval (CI) for model testing. All these analyses were performed using SPSS 24.0.

## 3 Results

### 3.1 Scale validation

The EFA in subsample 1 (*n* = 633) supported a three-factor structure for the Chinese version of the MSES. Compared with the four-factor and other competing models, the three-factor model demonstrated the best overall model fit, with fit indices of CFI = 0.974, TLI = 0.951, RMSEA = 0.080, and SRMR = 0.020. The oblique Geomin-rotated factor loadings ranged from 0.428 to 0.983. No items were excluded, as all factor loadings exceeded the recommended threshold of 0.40.

**Figure 2:**
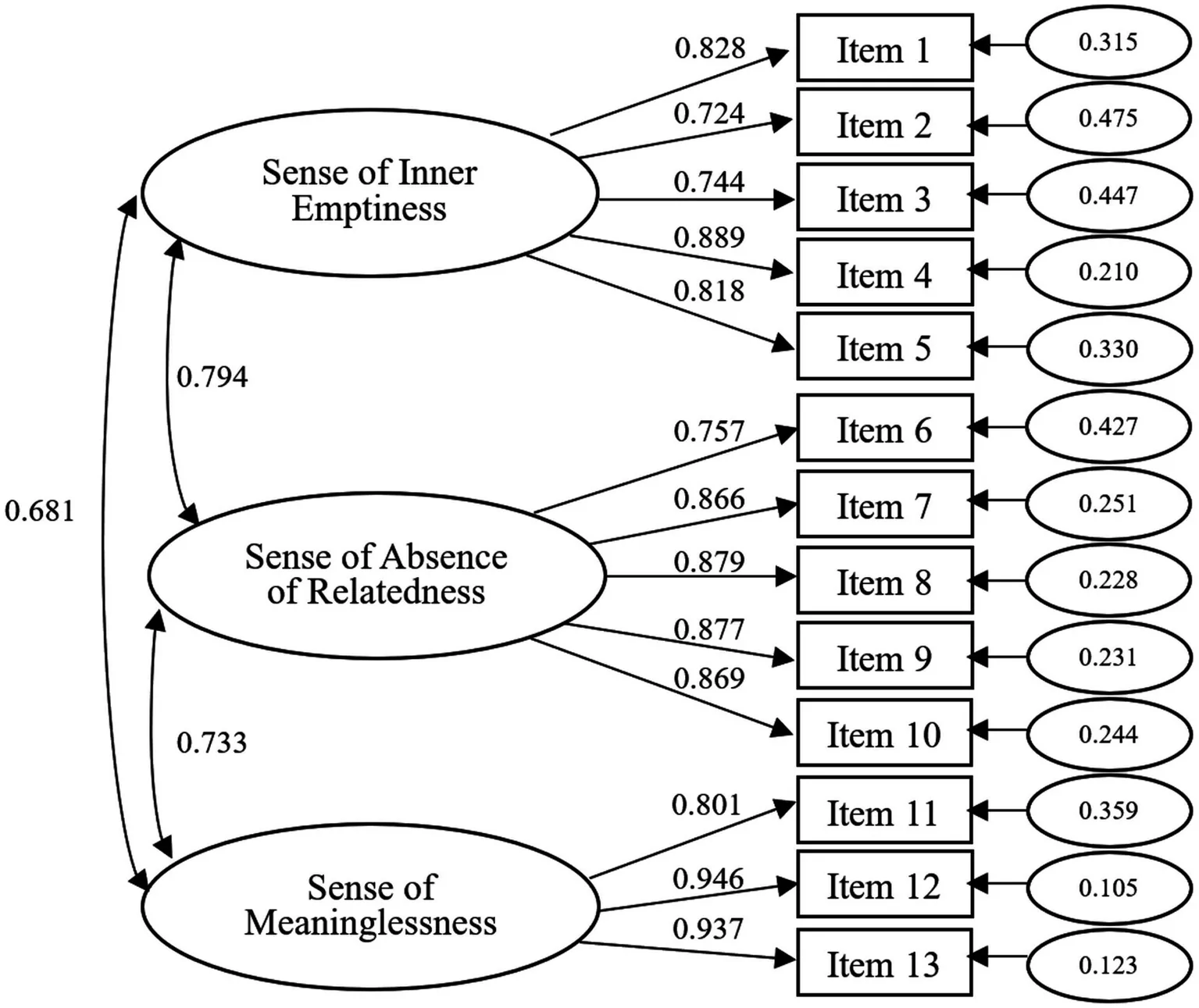
Factor structure of the Chinese version of the Multidimensional Sense of Emptiness Scale.

The CFA in the independent subsample 2 (*n* = 633) cross-validated the three-factor structure extracted via EFA from the separate exploratory subsample 1, yielding mixed model fit indices: CFI = 0.958, TLI = 0.954, and SRMR = 0.053 met the established good-fit thresholds, whereas RMSEA = 0.090 slightly exceeded the pre-specified cutoff of 0.08. Taken together, the fit indices offer preliminary supportive evidence for the plausibility of the three-factor structure of the Chinese MSES. Figure 2 illustrates the factor structure of the MSES as applied among Chinese university students.

The Chinese version of the MSES and its three subscales demonstrated excellent internal consistency reliability, with Cronbach’s *α* values of 0.901, 0.925, 0.912, and 0.950, respectively.

### 3.2 Participants’ characteristics

The average age of participants was 21.82 years (standard deviation: 3.15), with female participants having a significantly higher mean age than those of other genders (*p* < 0.001), as shown in Table 1. Significantly fewer females were only children in their families (41.49%, *p* < 0.001). A higher percentage of participants identifying as other genders reported low family monthly income (24.32%) or high family monthly income (18.92%) (*p* < 0.001), while the majority of male and female participants had family incomes in a modest range (60.3 and 65.96%, respectively). Additionally, a higher percentage of participants identifying as other genders reported having a religious belief (13.51%, *p* < 0.001), and a greater percentage of male participants were single (78.35%, *p* < 0.01). Overall, 26.5% of participants reported experiencing clinically significant depressive symptoms.

**Table 1:** Sociodemographic information and scores of study variables.

Categories	Total *n* = 1,266		Males *n* = 665		Females *n* = 564		Others *n* = 37		*p*
	*n*	%	*n*	%	*n*	%	*n*	%	
Age: mean ± SD	21.82 ± 3.15		21.34 ± 3.12		22.42 ± 3.04		21.30 ± 3.81		0.000
Only-child									0.000
Yes	637	50.32%	380	57.14%	234	41.49%	23	62.16%	
No	629	49.68%	285	42.86%	330	58.51%	14	37.84%	
Family monthly income									0.000
Below 3,000 RMB	103	8.14%	52	7.82%	42	7.45%	9	24.32%	
3,000–9,999 RMB	347	27.41%	170	25.6%	170	30.14%	7	18.92%	
10,000–49,999 RMB	440	34.76%	231	34.7%	202	35.82%	7	18.92%	
Above 50,000 RMB	85	6.71%	42	6.32%	36	6.38%	7	18.92%	
Religion									0.000
Have a religion	29	2.29%	12	1.80%	12	2.13%	5	13.51%	
Have no religion	1,237	97.71%	653	98.20%	552	97.87%	32	86.49%	
Relationship status									0.004
Single	943	74.49%	521	78.35%	395	70.04%	27	72.97%	
Not single	323	25.51%	144	21.65%	169	29.96%	10	27.03%	
Perceived support	10.66 ± 2.54		10.76 ± 2.57		10.66 ± 2.42		8.74 ± 3.26		0.000
Family support	5.29 ± 1.44		5.42 ± 1.40		5.22 ± 1.42		4.11 ± 1.82		0.000
Friend support	5.37 ± 1.37		5.34 ± 1.41		5.44 ± 1.29		4.63 ± 1.63		0.002
Spiritual emptiness	23.63 ± 9.31		24.24 ± 9.54		22.57 ± 8.85		28.81 ± 9.71		0.000
Inner emptiness	9.72 ± 4.04		10.08 ± 4.14		9.14 ± 3.78		12.05 ± 4.51		0.000
Absence of relatedness	8.64 ± 3.81		8.81 ± 3.88		8.29 ± 3.63		10.86 ± 4.13		0.000
Meaninglessness	5.27 ± 2.46		5.35 ± 2.52		5.14 ± 2.36		5.89 ± 2.76		0.103
Depression	6.95 ± 5.47 (26.5%)		6.75 ± 5.69 (26.5%)		6.97 ± 5.04 (24.8%)		10.08 ± 6.99 (51.4%)		0.001

Participants identifying as other genders perceived significantly less support than male and female participants, including less family support (*p* < 0.001), friend support (*p* < 0.01), and total perceived support (*p* < 0.001). Those identifying as other genders also experienced significantly higher levels of spiritual emptiness (*p* < 0.001) than male and female participants, including in the subscales of inner emptiness (*p* < 0.001) and absence of relatedness (*p* < 0.001). Furthermore, participants identifying as other genders suffered from significantly higher levels and percentages of depressive symptoms compared to male and female university students (*p* < 0.01). Male participants perceived the most family support (mean: 5.42 vs. 5.22 for females and 4.11 for other genders) but also reported a higher level of spiritual emptiness compared to female participants (mean: 24.24 vs. 22.57), including in the subscales of inner emptiness (10.08 vs. 9.14) and absence of relatedness (8.81 vs. 8.29).

### 3.3 Correlation and regression tests

Pearson’s correlation tests indicated that perceived support, spiritual emptiness, and depression were significantly related (*r* = 0.42–0.69, all *p* < 0.001). As shown in Table 2, family income was positively correlated with perceived support (*β* = 0.42, *p* < 0.001) and negatively correlated with spiritual emptiness (*β* = −1.27, *p* < 0.01) and depressive symptoms (*β* = −0.63, *p* < 0.01), although the relationships between family income and both spiritual emptiness and depression became insignificant after including perceived support (*p* > 0.05). Age (*β* = −0.41, *p* < 0.001) and relationship status (*β* = −2.02, *p* < 0.01) were distinctively negatively associated with spiritual emptiness when considering the relationship of perceived support, indicating that younger university students in China experienced higher levels of spiritual emptiness than older students, and that single university students experienced greater spiritual emptiness than those who were not single. Additionally, gender had a significantly positive relationship with depressive symptoms (*β* = 0.72, *p* < 0.01), indicating that females and participants identifying as other genders experienced higher levels of depressive symptoms than males.

**Table 2:** Regression models.

Coefficient	Perceived support	Spiritual emptiness		Depression		
	Model 1	Model 1	Model 2	Model 1	Model 2	Model 3
Constant	8.71 (1.22)***	41.89 (4.46)***	55.52 (4.13)***	10.50 (2.67)***	17.91 (2.53)***	−3.64 (2.13)
Age	0.00 (0.03)	−0.41 (0.10)***	−0.41 (0.09)***	−0.08 (0.06)	−0.08 (0.05)	0.08 (0.42)
Gender	−0.20 (0.15)	−0.24 (0.54)	−0.55 (0.49)	0.68 (0.32)*	0.51 (0.30)	0.72 (0.23)**
Relationship status	0.07 (0.19)	−2.14 (0.70)**	−2.02 (0.64)**	−0.29 (0.42)	−0.23 (0.39)	0.55 (0.30)
Family income	0.42 (0.10)***	−1.27 (0.38)**	−0.62 (0.35)	−0.63 (0.23)**	−0.27 (0.21)	−0.03 (0.16)
Religion	0.61 (0.51)	−1.13 (1.85)	−0.17 (1.67)	−0.17 (1.11)	0.36 (1.02)	0.42 (0.79)
Only-child	−0.14 (0.17)	−0.36 (0.61)	−0.57 (0.55)	−0.16 (0.36)	−0.28 (0.34)	−0.06 (0.26)
Perceived support			−1.56 (0.11)***		−0.85 (0.07)***	−0.24 (0.06)***
Spiritual emptiness						0.39 (0.02)***
*R* ^2^	0.02	0.05	0.23	0.02	0.16	0.50
*F*	3.88**	8.70***	40.07***	2.46*	27.00***	123.35***

Standard errors in parentheses; *p < 0.05; **p < 0.01; ***p < 0.001. All categorical demographic covariates were recoded into multiple binary dummy variables with predefined reference groups (detailed in the Data Analysis section): gender (male as reference), relationship status (single as reference), family monthly income (below 3,000 RMB as reference), religious belief (having religious belief as reference), only-child status (only child as reference). Each reported coefficient for a categorical variable represents the pooled linear predictive effect of its full set of dummy contrasts on the corresponding outcome variable.

Controlling for the association of six sociodemographic factors, perceived support was significantly negatively correlated with spiritual emptiness (*β* = −1.56, *p* < 0.001) and depression (*β* = −0.85, *p* < 0.001) in model 2. In Model 3, perceived support (*β* = −0.24, *p* < 0.001) and spiritual emptiness (*β* = 0.39, *p* < 0.001) together played substantial roles in depression while controlling for the association of sociodemographic factors.

### 3.4 A moderated mediation model

**Figure 3:**
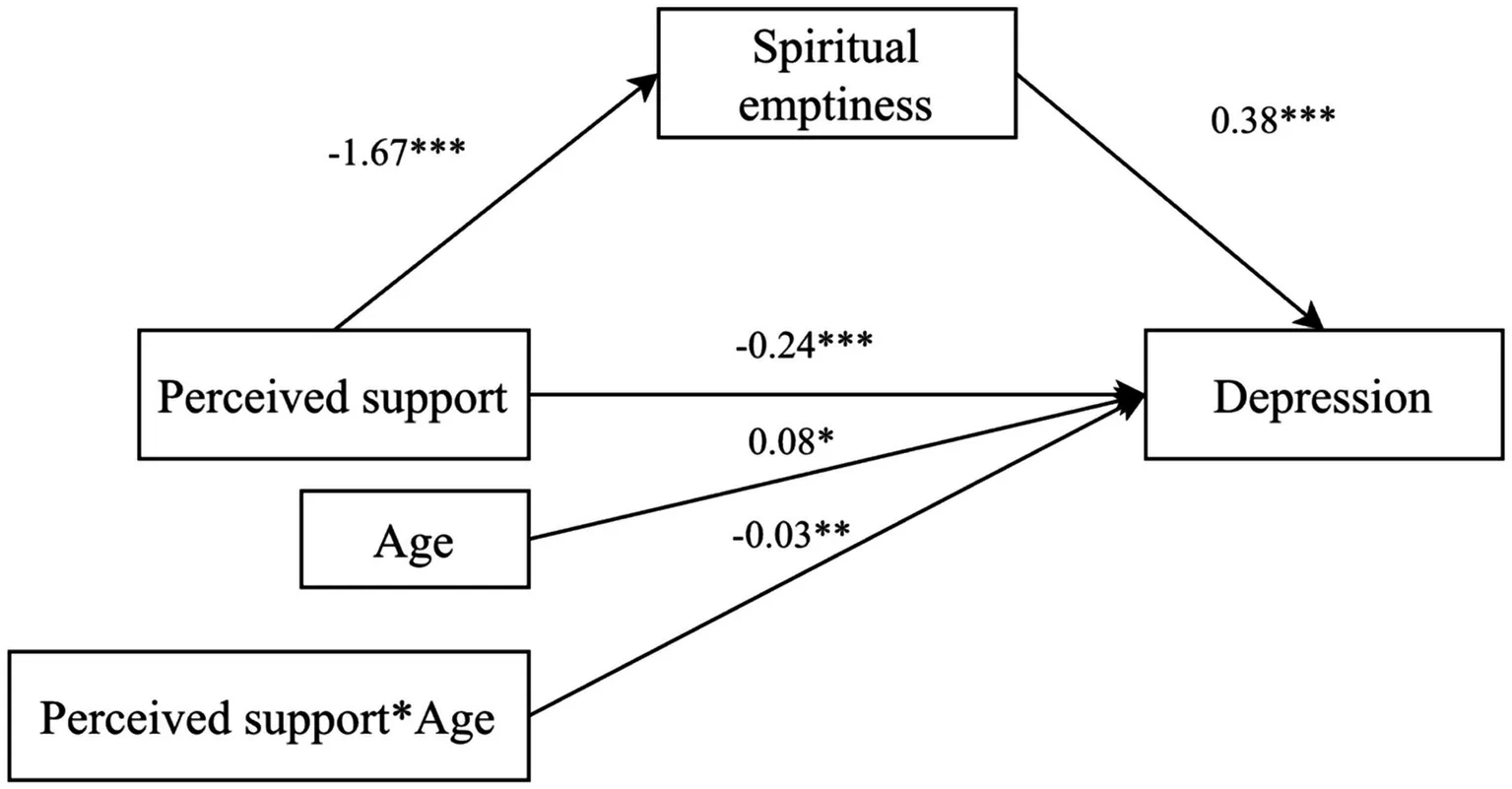
The final moderated mediation model. *p < 0.05; **p < 0.01; ***p < 0.001.

Table 3 presents the results of the moderated mediation analysis examining the role of age in moderating the association between perceived support and depression, as well as the mediation of spiritual emptiness in this relationship, while controlling for gender and relationship status. Based on the results, a final moderated mediation model (Figure 3) was specified.

**Table 3:** Conditional process analysis.

Model	Coeff.	SE	*t*	*p*	LLCI	ULCI
Mediator variable model						
Constant	28.95	0.90	32.10	0.000	27.18	30.72
Perceived support → emptiness	−1.67	0.09	−18.49	0.000	−1.85	−1.50
Gender → emptiness	−0.93	0.42	−2.24	0.026	−1.75	−0.11
Relationship status → emptiness	−3.12	0.53	−5.90	0.000	−4.16	−2.08
Dependent variable model						
Constant	−3.76	0.58	−6.44	0.000	−4.90	−2.61
Perceived support → depression	−0.24	0.05	−4.97	0.000	−0.34	−0.15
Emptiness → depression	0.38	0.01	28.39	0.000	0.36	0.41
Age → depression	0.08	0.04	2.18	0.029	0.01	0.15
Perceived support × age → depression	−0.03	0.01	−2.86	0.004	−0.06	−0.01
Gender → depression	0.66	0.20	3.32	0.001	0.27	1.05
Relationship status → depression	0.51	0.27	1.91	0.057	−0.01	1.03
Conditional direct effects moderated by age						
Mean-1SD	−0.13	0.06	−2.08	0.038	−0.26	−0.01
Mean	−0.24	0.05	−4.97	0.000	−0.34	−0.15
Mean+1SD	−0.35	0.06	−5.84	0.000	−0.47	−0.23
Indirect effect via spiritual emptiness						
Perceived support → emptiness → depression	−0.64	0.06			−0.75	−0.54

*N* = 1,266; PROCESS for SPSS Model 5 results; bootstrap size = 5,000; Coeff., coefficient; SE, standard error; LL, lower limit; UL, upper limit; CI, 95% confidence interval; SD, standard deviation. The presented gender coefficient represents the combined dummy contrast comparing gender minority participants (other genders) against male participants (reference group); the relationship status coefficient reflects the single binary dummy contrast of non-single participants relative to single participants (reference group).

As shown in Figure 3, perceived support was significantly and negatively associated with spiritual emptiness (*β* = −1.67, *p* < 0.001), which, in turn, exhibited a significant positive association depressive symptoms (*β* = 0.38, *p* < 0.001), thereby supporting H1 and H2. These findings support the hypothesized indirect pathway from perceived support to depression via spiritual emptiness. The statistical direct effect of perceived support on depression remained significant (*β* = −0.24, *p* < 0.001). Additionally, as indicated in Table 3, the indirect effect of perceived support on depression via spiritual emptiness (*β* = −0.64) was significant, with a 95% CI of (−0.75, −0.54), indicating a robust mediating mechanism and providing support for H3.

**Figure 4:**
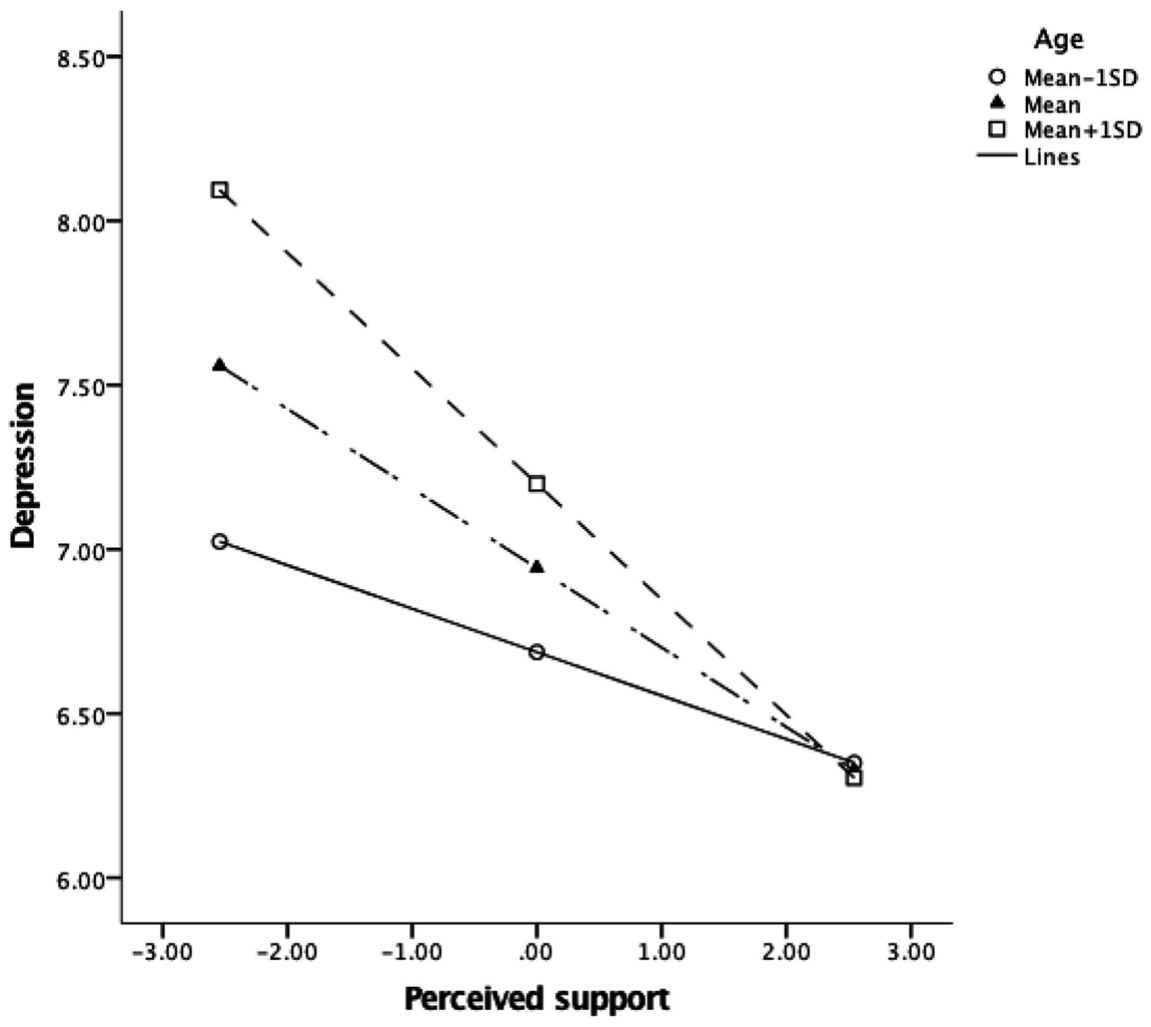
Age as a moderator in the association between perceived support and depression.

Regarding the moderating effect, both Table 3 and Figure 4 illustrate that age significantly moderated the relationship between perceived support and depression (*β* = −0.03, *p* < 0.01). Simple slope analyses indicated that the negative association was stronger among participants aged +1 SD above the mean (*β* = −0.35, *p* < 0.001) compared with those aged −1 SD below the mean (*β* = −0.13, *p* < 0.05), thereby supporting H4.

Gender and relationship status were included as covariates in both the mediator and outcome models. As shown in Table 3, in the mediator model, gender was significantly associated with spiritual emptiness (*β* = −0.93, *p* < 0.05), and relationship status was also significantly associated with spiritual emptiness (*β* = −3.12, *p* < 0.001), indicating lower levels of spiritual emptiness among participants who were not single. In the outcome model, gender was significantly correlated with depressive symptoms (*β* = 0.66, *p* < 0.01), while relationship status showed a marginal association with depressive symptoms (*β* = 0.51, *p* = 0.057).

## 4 Discussion

This study tested the reliability and validity of the Chinese adaptation of the MSES and revealed the correlational patterns among perceived support, spiritual emptiness, and depression. The results supported the consistency and precision of the MSES in assessing spiritual emptiness among Chinese college students, supporting its original three-factor structure in our Chinese version. As a newly-developed instrument with an inclusive theoretical basis, the MSES can be reliably validated for examining spiritual emptiness among university students and the general populations (Ermis-Demirtas et al., 2022).

In addition to scale validation, this study found that a substantial proportion of Chinese university students reported depressive symptoms, which is consistent with previous reports indicating a prevalence of approximately one-quarter (Psychology China, 2023). Furthermore, perceived support was negatively associated with both spiritual emptiness and depressive symptoms, while spiritual emptiness partially mediated the relationship between perceived support and depressive symptoms. These findings support our hypotheses and are generally consistent with previous studies demonstrating that perceived social support is an important protective factor against spiritual emptiness and depressive symptoms among college students. Existing evidence suggests that support from parents and peers can reduce feelings of loneliness and hopelessness, thereby buffering against spiritual emptiness among young adults (Bareket-Bojmel et al., 2021; Mahon and Yarcheski, 2017; Pamukçu and Meydan, 2010). In addition, supportive relationships foster a stronger sense of connectedness and meaning in life, both of which have been associated with lower levels of depression and better psychological wellbeing (Liu et al., 2022; Ryan and Solky, 1996; Zuo et al., 2021). Importantly, our findings extend the existing literature by suggesting that perceived support is negatively associated with depressive symptoms not only directly but also indirectly by reducing individuals’ sense of spiritual emptiness. One possible explanation is that supportive relationships satisfy individuals’ fundamental need for relatedness and promote a greater sense of meaning and purpose in life. Consequently, individuals who perceive higher levels of social support may be less vulnerable to spiritual emptiness, which is positively related to depressive symptoms. Therefore, interventions aimed at strengthening perceived support may help reduce spiritual emptiness and depressive symptoms among university students.

Age was found to moderate the association between perceived support and depressive symptoms, with the negative correlational linkage between perceived support and depressive symptoms being more pronounced among older university students. This finding differs from a previous community-based study involving adults across a wide age range, which reported that the protective effect of social support was stronger among younger participants than among older adults (Segrin, 2003). One possible explanation for this discrepancy is the difference in study populations. Unlike the previous study, which compared individuals across the lifespan, our sample was restricted to university students. Within this developmental stage, older adults may have developed more instrumental support, expressive support, and closer confidant relationships than younger adults, which may strengthen their resilience to depression (Dean et al., 1981). In contrast, younger college students often experience multiple challenges related to study and living transitions, including separation from family and friends, adjustment to independent living, and the need to establish new support networks (Credé and Niehorster, 2012; Lafreniere and Ledgerwood, 1997; Denovan and Macaskill, 2013). These challenges may reduce both the availability and effectiveness of perceived support in buffering against depressive symptoms during the early years of university.

Regarding the influence of covariates, university students who were not single reported lower levels of spiritual emptiness than single students. This finding is consistent with previous studies suggesting that being in a romantic relationship is associated with better psychological wellbeing among young adults (Adamczyk et al., 2021). Compared with students in relationships, single university students may experience greater loneliness and receive less emotional support and companionship from a significant other, which may contribute to higher levels of spiritual emptiness (Adamczyk, 2016). In addition, students identifying as other genders perceived lower levels of social support and reported higher levels of spiritual emptiness and depressive symptoms than their cisgender counterparts, which is consistent with previous research (He et al., 2024; Hunter et al., 2021; Jalene et al., 2022; Lefevor et al., 2019; Reisner et al., 2016; Ross-Reed et al., 2019). One possible explanation is that limited public understanding of sexual and transgender issues among the general population may reduce the availability of support from family and friends (Koken et al., 2009; Galupo et al., 2014). Furthermore, these students may feel guilty and perceive themselves as “abnormal” when they fail to conform to the prevailing culture of heteronormativity and filial piety, leading to feelings of meaninglessness, loneliness, and emptiness, which may increase their vulnerability to poor mental health outcomes, particularly depression (He et al., 2024; Hendricks and Testa, 2012; Sun et al., 2020; Kittiteerasack et al., 2022; Rogowska and Cisek, 2024).

In our study, a higher proportion of female students than male students came from families with more than one child, despite all participants having been born during China’s one-child policy era (before 2016). This pattern may reflect the combined influence of traditional son preference and fertility policies in China (Liu and Jiang, 2021). Previous studies have shown that, under the influence of son preference, some families continued childbearing after the birth of a daughter in the hope of having a son (Basu and de Jong, 2010). In addition, exemptions to the one-child policy under specific circumstances, such as for families whose first child was a daughter, may also have contributed to this pattern (Gu et al., 2007).

The current study holds significance both theoretically and practically. Theoretically, we expanded the generalizability of the MSES into the Chinese population and proposed a framework on the relationship between perceived support, depression, and spiritual emptiness. Future researchers are encouraged to investigate spiritual emptiness among young people using the MSES and our conceptual framework. Practically, since perceived support was negatively related to spiritual emptiness and depressive symptoms, future researchers could develop relevant interventions, such as supportive social work groups, to enhance college students’ perceived support and mitigate adverse outcomes, given the high depression rates (Chia et al., 2008; Segal-Engelchin et al., 2015).

Several limitations inherent in this study warrant careful consideration. Primarily, the utilization of a cross-sectional research design inherently restricts the capacity to examine the dynamic relationships and potential causal pathways among perceived support, spiritual emptiness, and depressive symptoms for college students. Therefore, longitudinal or qualitative studies should be conducted to further substantiate the causal relationships among these three variables. Secondly, although the study achieved a large sample size and identified gender differences, the proportion of participants identifying as other gender types was significantly smaller than that of cisgender participants, which may limit the generalizability of the findings regarding gender minority students in China. Moreover, despite the large sample size and its adequacy for the analyses performed, participants were recruited from only a limited number of universities in China. Therefore, the generalizability of the findings should be interpreted with caution, as cultural and regional differences across universities and geographical areas may influence the characteristics of university students and, consequently, the associations among perceived support, spiritual emptiness, and depressive symptoms. Thirdly, information regarding prior psychiatric diagnoses, mental health treatment, and neurodevelopmental conditions was not collected. Given that these factors may influence perceived support, spiritual emptiness, and depressive symptoms, future research should assess and control for these characteristics to reduce potential confounding effects and provide a more comprehensive understanding of the relationships among the study variables. Lastly, as all core variables were collected using self-report measures at a single time point, the possibility of common method bias cannot be fully excluded. Future studies should adopt multi-wave or multi-method designs to further validate the robustness of the proposed model.

## 5 Conclusion

In conclusion, this study provides evidence for the reliability and validity of the Chinese version of the MSES and supports its applicability for assessing spiritual emptiness among Chinese university students. The findings further demonstrate that perceived support is significantly associated with lower levels of spiritual emptiness and depressive symptoms, with spiritual emptiness serving as a partial mediator in this relationship. In addition, age was found to moderate the association between perceived support and depressive symptoms, indicating that the negative correlational linkage between perceived support and depressive symptoms was more pronounced among older university students.

Overall, the results highlight the importance of social support in mitigating psychological distress among university students and underscore spiritual emptiness as a meaningful psychological mechanism underlying this association. These findings suggest that interventions aimed at enhancing perceived social support may be beneficial in reducing both spiritual emptiness and depressive symptoms.

Future research should further examine the proposed model using longitudinal and multi-method designs to better clarify the temporal and potential causal relationships among perceived support, spiritual emptiness, and depression. In addition, future studies are encouraged to recruit more balanced samples, particularly increasing the representation of gender minority groups, and to collect and control for relevant clinical background variables, such as prior psychiatric diagnoses, mental health treatment history, and neurodevelopmental conditions, to reduce potential confounding effects.

## Data Availability

The raw data supporting the conclusions of this article will be made available by the authors, without undue reservation.
